# Safety and Efficacy of Topical Chitogel- Deferiprone-Gallium Protoporphyrin in Sheep Model

**DOI:** 10.3389/fmicb.2018.00917

**Published:** 2018-05-11

**Authors:** Mian L. Ooi, Katharina Richter, Amanda J. Drilling, Nicky Thomas, Clive A. Prestidge, Craig James, Stephen Moratti, Sarah Vreugde, Alkis J. Psaltis, Peter-John Wormald

**Affiliations:** ^1^Department of Surgery- Otolaryngology, Head and Neck Surgery, Basil Hetzel Institute for Translational Health Research, The University of Adelaide, Adelaide, SA, Australia; ^2^Adelaide Biofilm Test Facility, Sansom Institute for Health Research, University of South Australia, Adelaide, SA, Australia; ^3^School of Pharmacy and Medical Sciences, University of South Australia, Adelaide, SA, Australia; ^4^Clinpath Laboratories, Adelaide, SA, Australia; ^5^Department of Chemistry, Otago University, Dunedin, New Zealand

**Keywords:** chronic rhinosinusitis, *Staphylococcus aureus*, biofilm, Chitogel, Deferiprone, Gallium Protoporphyrin, topical agents, antimicrobial therapy

## Abstract

**Objectives:** Increasing antimicrobial resistance has presented new challenges to the treatment of recalcitrant chronic rhinosinusitis fuelling a continuous search for novel antibiofilm agents. This study aimed to assess the safety and efficacy of Chitogel (Chitogel®, Wellington New Zealand) combined with novel antibiofilm agents Deferiprone and Gallium Protoporphyrin (CG-DG) as a topical treatment against *S. aureus* biofilms *in vivo*.

**Methods:** To assess safety, 8 sheep were divided into two groups of 7 day treatments (*n* = 8 sinuses per treatment); (1) Chitogel (CG) with twice daily saline flush, and (2) CG-DG gel with twice daily saline flush. Tissue morphology was analyzed using histology and scanning electron microscopy (SEM). To assess efficacy we used a *S. aureus* sheep sinusitis model. Fifteen sheep were divided into three groups of 7 day treatments (*n* = 10 sinuses per treatment); (1) twice daily saline flush (NT), (2) Chitogel (CG) with twice daily saline flush, and (3) CG-DG gel with twice daily saline flush. Biofilm biomass across all groups was compared using LIVE/DEAD BacLight stain and confocal scanning laser microscopy.

**Results:** Safety study showed no cilia denudation on scanning electron microscopy and no change in sinus mucosa histopathology when comparing CG-DG to CG treated sheep. COMSTAT2 assessment of biofilm biomass showed a significant reduction in CG-DG treated sheep compared to NT controls.

**Conclusion:** Results indicate that CG-DG is safe and effective against *S. aureus* biofilms in a sheep sinusitis model and could represent a viable treatment option in the clinical setting.

## Introduction

Recalcitrant chronic rhinosinusitis is a difficult clinical entity to manage. Bacterial biofilms contribute to disease recalcitrance and have been shown to be associated with more severe disease (Bendouah et al., 2006; Psaltis et al., 2008; Singhal et al., 2010, 2011). Although oral antibiotics are frequently ineffective against biofilms (Costerton, 1995), it remains the only option available to achieve symptom control for many recalcitrant patients. However, with the growing prevalence of resistance to first-line antibiotics (World Health Organization, 2016) and the lack of research and development of new antibiotics (Conly and Johnston, 2005; World Health Organization, 2017), novel topical anti-biofilm agents are needed to help improve the outcomes in these patients.

Richter et al. first described the potent synergistic antimicrobial properties of Deferiprone and Gallium Protoporphyrin (DG) (Richter et al., 2016, 2017a,b). This agent targets the iron metabolism that is crucial for bacterial growth and survival (Braun, 2001; Weinberg, 2009). Deferiprone is an iron chelator approved by the U.S. Food and Drug Administration to treat thalassaemia major. Gallium Protoporphyrin IX is a heme analog with strong antibacterial activity against gram-positive bacteria, gram-negative bacteria and mycobacteria (Stojiljkovic et al., 1999; Hijazi et al., 2017). Gallium Protoporphyrin IX has been shown to kill *S. aureus* and Methicillin Resistant *S. aureus* (MRSA) in planktonic, biofilm and small colony variant form and has been shown to enhance the antimicrobial properties of commonly used antibiotics (Richter et al., 2017c). Deferiprone is thought to chelate iron from the bacteria's surrounding environment, forcing the bacteria to upregulate their iron transporter proteins. Deferiprone-dependent increased expression of iron transporter proteins are thought to enhance Gallium Protoporphyrin IX uptake into bacterial cells, thereby augmenting bacterial killing efficacy (Richter et al., 2016, 2017b). Consequently, the synergistic antimicrobial effects are observed mainly when Deferiprone and Gallium Protoporphyrin IX are given consecutively (Richter et al., 2016).

In this study, DG is incorporated within Chitogel (chitosan and dextran), a surgical hydrogel FDA approved for the use after sinus surgery, which acts as a drug carrier, that can be applied topically to fill the sinus cavities. The gel has been shown to allow the immediate and complete release of Deferiprone whilst Gallium Protoporphyrin IX is released more slowly (Richter et al., 2017b). This topical application allows higher concentration of drugs to be used for a localized action with less systemic side effects. The mucoadhesive properties of the hydrogel also increases contact time of these topical agents with the sinus mucosa and biofilms (Illum et al., 1994; Nakamura et al., 1999) augmenting its anti-biofilm effects.

The aims of this study were to assess the safety of CG-DG on healthy sinus mucosa and evaluate its efficacy as an anti-biofilm agent in a previously validated *S. aureus* biofilm-induced sheep sinusitis model.

## Materials and methods

This study was approved by the Animal Ethics Committee of both The University of Adelaide and the South Australian Health and Medical Research Institute (SAHMRI).

### Animals

Twenty three male merino sheep between 2 and 4 dental age (1–2 years of age) were used. All animals were drenched to eradicate the parasite *Oestrus Ovis*. Fifteen sheep were allocated to the efficacy arm and 8 to the safety arm. For the efficacy arm 5 sheep were randomized to each efficacy group (i) Twice daily saline flush (NT), (ii) Chitogel (CG) and (iii) Chitogel- Deferiprone-Gallium Protoporphyrin (CG-DG). For the safety arm, 4 sheep were randomized to each safety group (i) Chitogel (CG) and (ii) Chitogel- Deferiprone-Gallium Protoporphyrin (CG-DG).

### Bacterial inoculum

A known biofilm-forming reference strain of S. *aureus*, American Type Culture Collection (ATCC) 25923, was supplied by the Department of Microbiology, TQEH. A frozen glycerol stock was defrosted and subcultured overnight in 3 mL of nutrient broth (Oxoid, Adelaide, Australia) on a shaker at 37°C for 24 h before being transferred to a 1% nutrient agar plate (Oxoid). The plate was incubated at 37°C for 16–18 h, at which point a single colony forming unit (CFU) was diluted in 0.45% sterile saline to 0.5 McFarland standard and transferred on ice for instillation into sheep sinuses.

### Chitogel

The Chitogel is made up of a combination of three components; 5% succinyl-chitosan, 0.3% phosphate buffer and 3% dextran aldehyde (Chitogel^®^, Wellington, NZ). The components are manufactured and sterilized by Chitogel^®^. All stocks were stored at room temperature.

### Deferiprone and gallium protoporphyrin

Deferiprone (3-hydroxy-1,2-dimethylpyridin-4(1*H*)-one) (Sigma-Aldrich, St Louis, USA) and Gallium Protoporphyrin IX (Ga-PP IX) (Frontier Scientific, Logan, USA) were stored at room temperature.

### Preparation of chitogel

Dextran aldehyde (0.3 g) was dissolved in 10 mL of phosphate buffer then mixed with succinyl chitosan solution (0.5 g in 10 mL buffer) using sterile technique.

### Preparation of chitogel- deferiprone-gallium protoporphyrin

Deferiprone (20 mM) and Gallium Protoporphyrin (250 μg/mL) were diluted in 10 mL of phosphate buffer under sterile conditions the day before use. This prepared solution was then used to dissolve dextran aldehyde prior to mixing with 10 mL of succinyl chitosan using sterile techniques.

### Anaesthetic protocol

For every surgical procedure, all sheep underwent general anesthesia given by an experienced animal handler. Intravenous phenobarbitone was given at induction (19 mg/kg) and sheep were intubated and placed onto 1.5–2% inhalation isoflurane to maintain anesthesia. Each sheep was placed in a supine position on a wooden cradle and supported on a head ring with neck slightly flexed. Each nasal cavity was sprayed twice with Cophenylcaine Forte (ENT Technologies Pty Ltd., Australia) 10 min prior to any procedures.

### Surgical protocol

As per protocol all sheep underwent middle turbinectomy and anterior ethmoid complex resection, which is then followed by a 3–4 week convalescence period. Frontal trephination was later performed by placing mini trephines bilaterally on the sheep's forehead, 1cm lateral from the midline and along a line connecting the superior aspect of the orbital rims. The placement of trephines was confirmed when fluorescein flushed via trephines (0.1 mL diluted in 100 mL of physiological saline) was visualized to be draining from the frontal sinus ostium.

### Safety arm

In the safety arm, following frontal trephination, the gels were instilled into each sinus cavity via mini trephines until gel extrusion from the frontal sinus ostium was visualized under direct endoscopic view. The mini trephines were then capped. Gel instilled was left undisturbed within the sinus cavities for 24 h before beginning sinus irrigation via mini trephines with 15 mL of sterile physiological saline twice a day. On day 8, all safety sheep were euthanized and sinus mucosa harvested for histopathological and SEM analysis.

### Efficacy arm

In the efficacy arm, following frontal trephination the frontal ostia were packed with petroleum gauze (Vaseline, Kendall, Mansfield, MA). 1 mL of 0.5 McFarland Units of *S. aureus* was then instilled into each sinus cavity via mini trephines and capped. Bacterial biofilms were allowed to form over the next 7 days. On day 8, the petroleum gauze was removed and each sheep was randomly assigned into one of three efficacy groups (i) Twice daily saline flush (NT), (ii) Chitogel (CG) and (iii) Chitogel- Deferiprone-Gallium Protoporphyrin (CG-DG). For sheep assigned to gel groups (ii) and (iii), the gels were instilled into each sinus cavity via mini trephines until gel extrusion from the frontal sinus ostium was visualized under direct endoscopic view. The mini trephines were then capped. For all groups, sinuses were irrigated 24 h later with 15 mL of sterile physiological saline twice a day for the remaining 6 days of treatment. On day 8, all sheep were euthanized and sinus mucosa harvested for histopathological analysis and biofilm biomass imaging.

### Safety analysis

#### Histopathology evaluation

One 1 × 1 cm mucosal section from each sinus was fixed in 2% formalin solution and sent for histopathology preparation (Adelaide Pathology and Partners, Adelaide, Australia). Samples were embedded in paraffin and stained with hematoxylin & eosin. Microscopic evaluation of tissue damage and inflammation was performed by a pathologist blinded to all clinical data using light microscopy (Eclipse 90i, Nikon instruments Inc, Melville, NY).

#### Scanning electron microscopy evaluation

From each sinus, a sample of 5 × 5 mm tissue was obtained, sonicated in saline, then submerged in SEM fixative (4% paraformaldehyde/1.25% glutaraldehyde in PBS + 4% sucrose, pH 7.2) for at least 24 h. Tissues were washed in a washing buffer (PBS + 4% sucrose) for 5 min then post fixed in 2% OsO_4_ in water for 1 h. All samples underwent a graded dehydration of 70, 90, and 100% ethanol, then dried using hexamethyldisilazane (HMDS). Following that, all tissues were mounted on stubs and carbon coated. Images were taken using an XL30 Field Emission Gun Scanning Electron Microscope (Phillips, Eindhoven, Netherlands).

#### Quantification of plasma deferiprone and gallium protoporphyrin levels

Plasma samples were analyzed for Deferiprone and GaPP using high performance liquid chromatography (HPLC) on a Shimadzu UFLC XR (Shimadzu Cooperation, Kyoto, Japan). For the quantification of Deferiprone, 250 μl plasma was mixed with 750 μl methanol (HPLC grade, Merck, Darmstadt, Germany). The samples were vortexed for 1 min and centrifuged for 4 min at 14,800 rpm at room temperature (Eppendorf 5804R, Eppendorf, Hamburg, Germany). The clear supernatant (50 μl) was quantified on a Phenomenex Synergi 4 μm Fusion-RP LC column coupled to a security guard cartridge (Phenomenex, Lane Cove, NSW, Australia) using methanol/0.1 M orthophosphate buffer pH 7.2 (15%: 85%) as mobile phase at a flow rate of 2.0 ml/min. The Deferiprone concentration was detected at 280 nm and calculated against a standard curve ranging from 1.0 to 10.0 μg/ml Deferiprone (*R*^2^ > 0.992). For the quantification of GaPP, solid phase extraction (SPE) was performed using Oasis PRiME HLB cartridges 1 cc/30 mg (Waters, Dundas, NSW, Australia). Samples were prepared according to the manufacturer's protocol. Briefly, 250 μl plasma was mixed with 250 μl orthophosphoric acid (4%) and placed in a SPE cartridge. After washing with 5% methanol in Milli-Q water, 500 μl methanol was used to elute GaPP. The clear eluate (50 μl) was quantified using methanol/0.1 M orthophosphate buffer pH 7.2 (70%: 30%) as mobile phase at a flow rate of 1.0 ml/min. The GaPP concentration was detected at 405 nm and calculated against a standard curve ranging from 0.02 to 10.0 μg/ml GaPP (*R*^2^ > 0.995).

### Efficacy analysis

#### Biofilm biomass

Method of biofilm analysis were as described in previous studies (Ha et al., 2007; Singhal et al., 2012; Drilling et al., 2014; Paramasivan et al., 2014b; Rajiv et al., 2015). Two random 1 × 1 cm mucosal sections from each sinus were sampled. Each sample was briefly immersed in phosphate buffered solution to wash off planktonic cells and stained with LIVE/DEAD BacLight stain (Life Technology, Mulgrave, VIC, Australia) as per manufacturer's instructions. Biofilm biomass was assessed using confocal scanning laser microscope (LSM 710, Zeiss, Germany). Within each sample 3 of the areas with highest biofilm presence had axial Z stacks recorded to construct a 3D virtual image of the overlying tissue mucosa and biofilm, making a total of 6 Z-stack images per sinus. Eighty individual images of each representative area were taken as one Z stack image (Image properties: line average 4, 512 × 512 pixels, Z-stack 80 steps). The COMSTAT2 computer software (Lyngby, Denmark) was utilized to quantify biofilm biomass in each Z-stack (Heydorn et al., 2000; Klinger-Strobel et al., 2016).

#### Histopathology grading

One 1 × 1 cm mucosal section from each sinus was fixed in 2% formalin solution and sent for histopathology preparation (Adelaide Pathology and Partners). Samples were embedded in paraffin and stained with hematoxylin & eosin. Microscopic evaluation and tissue grading was performed by a pathologist blinded to all clinical data using light microscopy (Eclipse 90i, Nikon instruments Inc, Melville, NY). Degree of inflammation (lymphocytes, plasma cells, histiocytes and mast cells), acute inflammation (neutrophils), oedema, fibrosis and cilia were graded using an arbitrary scale (Boase et al., 2013; Drilling et al., 2014; Rajiv et al., 2015). Degree of inflammation, oedema and fibrosis were each graded from 0 to 3; 0 = none, 1 = mild, 2 = moderate, 3 = severe. Acute inflammation was graded from 0 to 2; 0 = none, 1 = mild, 2 = severe. Cilia were graded as minimal loss, focal loss, moderate loss, severe loss.

#### Statistical analysis

Comparison of mucosal biofilms between treatment groups were analyzed using Kruskal Wallis One-way analysis of variance (ANOVA) with Dunn's multiple comparison test. Comparison of histopathology grading between treatment groups in the efficacy arm were analyzed using Two-way analysis of variance (ANOVA) with Dunnett's multiple comparison test. Statistical significance was considered at *p* < 0.05. All statistical tests were done using GraphPad Prism 7.0b software (San Diego, CA).

## Results

### Safety arm

#### Histopathological analysis

**Figure 1:**
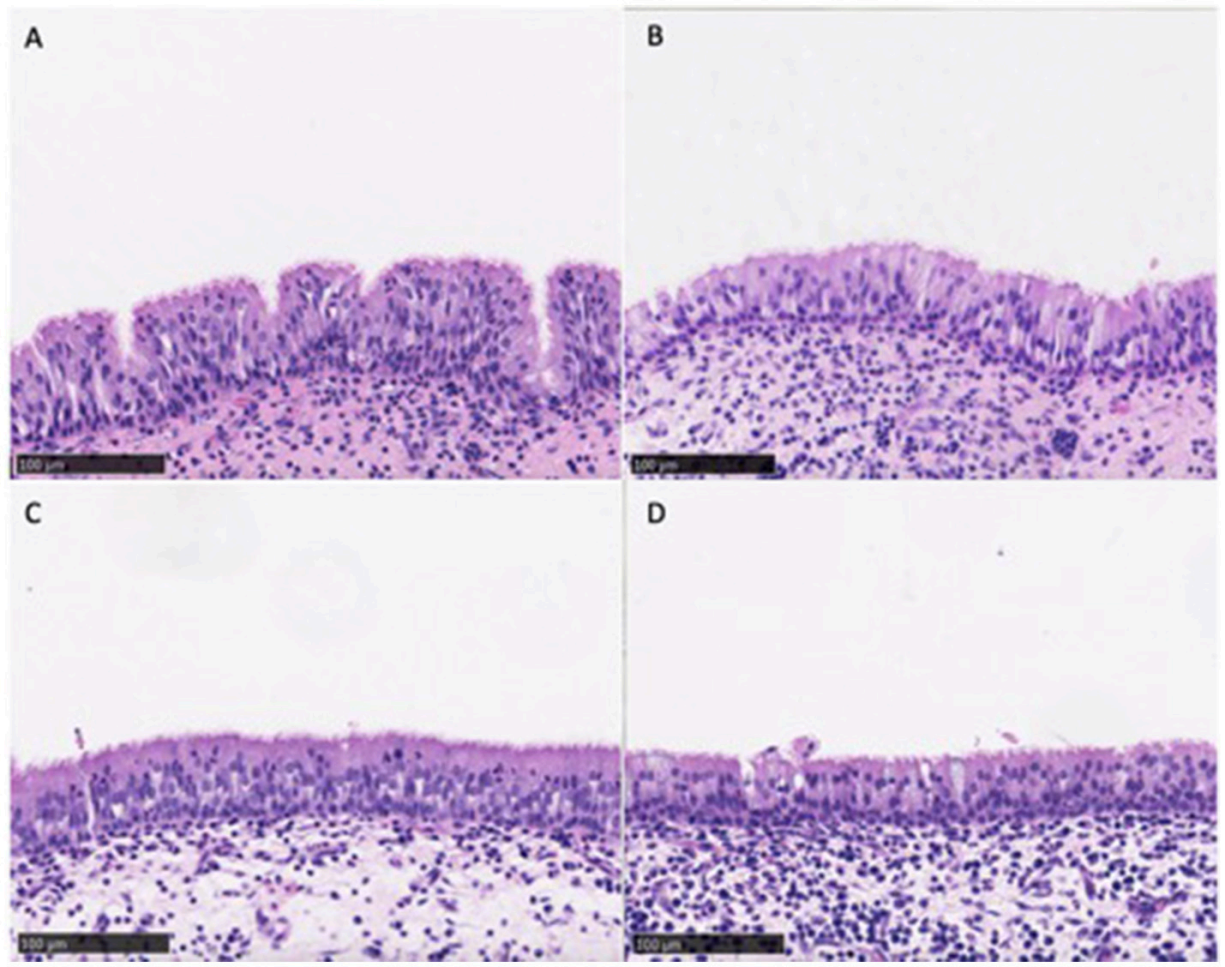
Representative images of sinus mucosa histology harvested from sheep in the safety arm treated with CD **(A,B)** and CD-DG **(C,D)**. All sinus mucosa showed pseudostratified columnar epithelial layer with no metaplasia, indicating that test treatments were safe for sinus topical application. CG, Chitogel; CG-DG, Chitogel- Deferiprone-Gallium Protoporphyrin.

Similar mucosal architecture was noted in all sinus samples obtained from CG and CG-DG treated groups, showing a pseudostratified columnar epithelial layer intersected with goblet cells. No squamous metaplasia of epithelium was identified in any samples (Figure 1). These images reflect that the test treatments are safe to apply topically to sinus mucosa.

#### SEM tissue analysis

**Figure 2:**
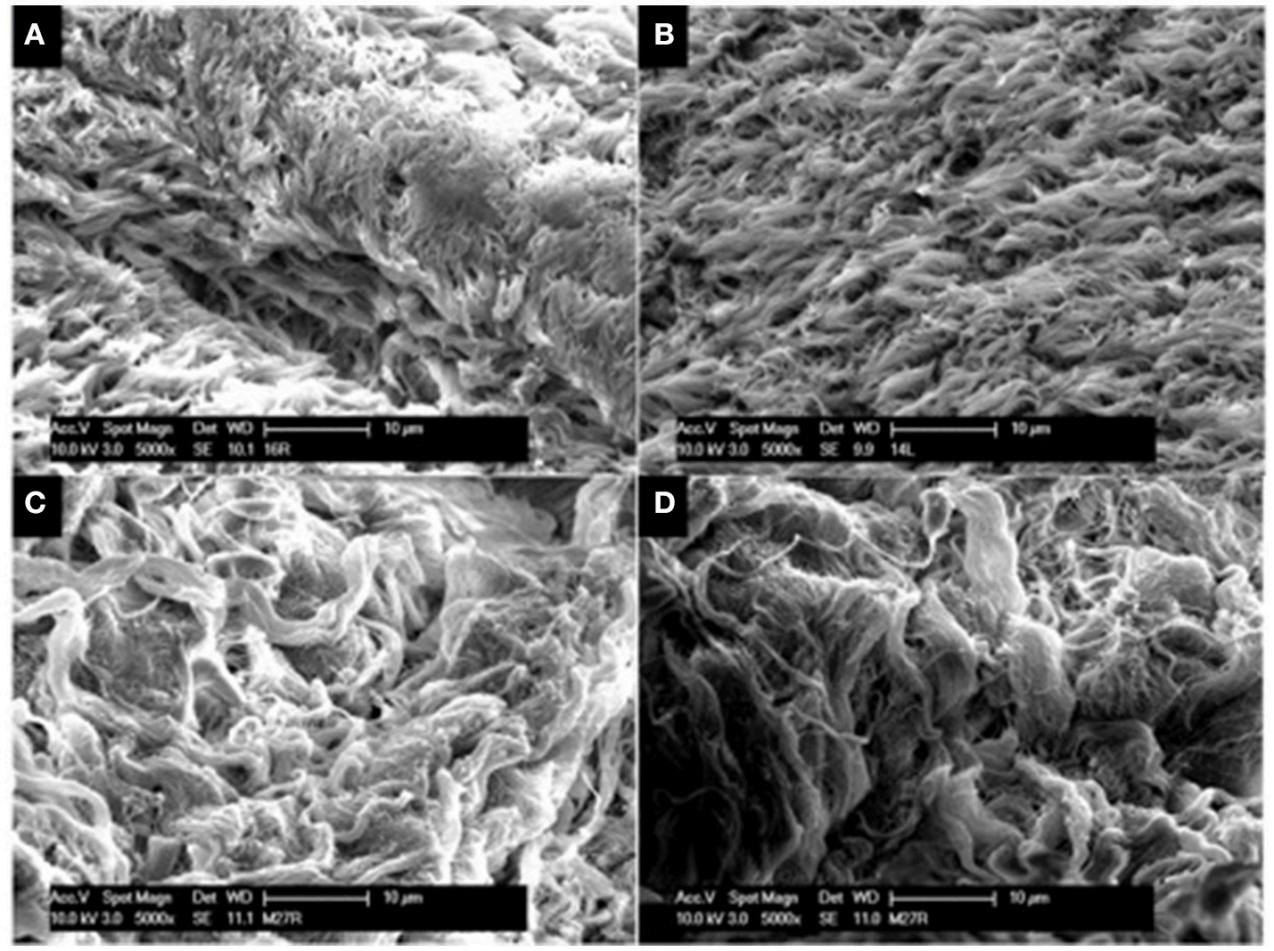
Representative SEM images of sinus mucosa harvested from sheep in the safety arm treated with CG gel **(A,B)** and CG-DG gel **(C,D)**. SEM allowed assessment for ciliary presence and morphology on sinus mucosa. No ciliary denudation were observed in both treated groups, indicating that test treatments were not ciliotoxic. CG, Chitogel; CG-DG, Chitogel- Deferiprone-Gallium Protoporphyrin.

SEM was employed to assess the presence and integrity of cilia present on sinus mucosal samples. In all sinus mucosal samples collected, there were no signs of ciliary denudation in both CG and CG-DG treated groups (Figure 2). These images reflect that the test treatments were not ciliotoxic on ciliated human respiratory cells.

#### Plasma deferiprone and gallium protoporphyrin levels

**Figure 3:**
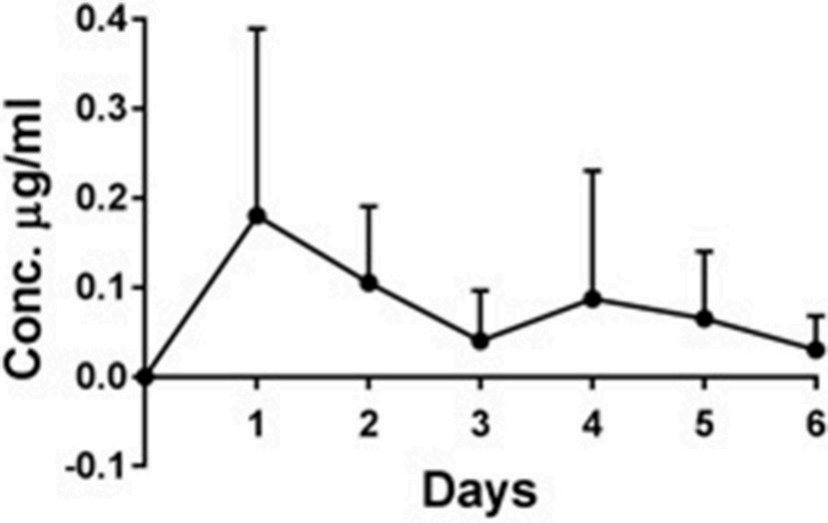
*In vivo* plasma concentration (μg/ml) ± standard deviation of deferiprone over 6 days, *n* = 4. Maximum Deferiprone plasma level of 0.18 μg/ml was detected at day 1 of topical application to sinuses, which is 110 times less than one oral dose of Deferiprone. No GaPP was detected in the plasma of all 4 sheep treated with CG-DG (data not shown). This indicates that CG-DG has negligible systemic effect from topical sinus application. GaPP, Gallium Protoporphyrin, CG, Chitogel; CG-DG, Chitogel- Deferiprone-Gallium Protoporphyrin.

The maximum Deferiprone concentration was reached after 1 day (0.18 μg/ml Deferiprone) in the 4 sheep treated with CG-DG (Figure 3). After 6 days the Deferiprone plasma concentration decreased to 0.03 μg/ml.

GaPP was not detected in the plasma of any of the 4 sheep treated with CG-DG (data not shown). According to the quantification level ranging from 0.02 to 10 μg/ml, this indicates a GaPP plasma concentration was below 0.02 μg/ml.

### Efficacy arm

#### Biofilm biomass analysis

**Figure 4:**
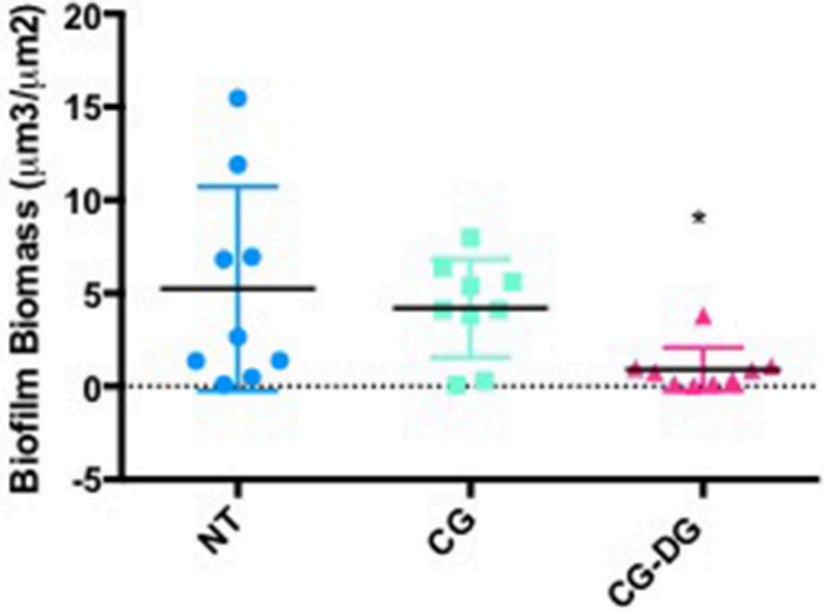
Scatter plots showing COMSTAT computation of *Staphylococcus aureus* biofilm biomass between **(A)** Twice daily saline flush (NT), **(B)** CG gel with twice-daily saline flush, and **(C)** CG-DG gel with twice-daily saline flush. Significant reduction of biofilm biomass seen in CG-DG treated group compared to NT and CG gel. *P < 0.05, Kruskal Wallis 1-way analysis of variance (ANOVA) with Dunn's multiple comparison test. NT, No treatment; CG, Chitogel; CG-DG, Chitogel- Deferiprone-Gallium Protoporphyrin.

**Figure 5:**
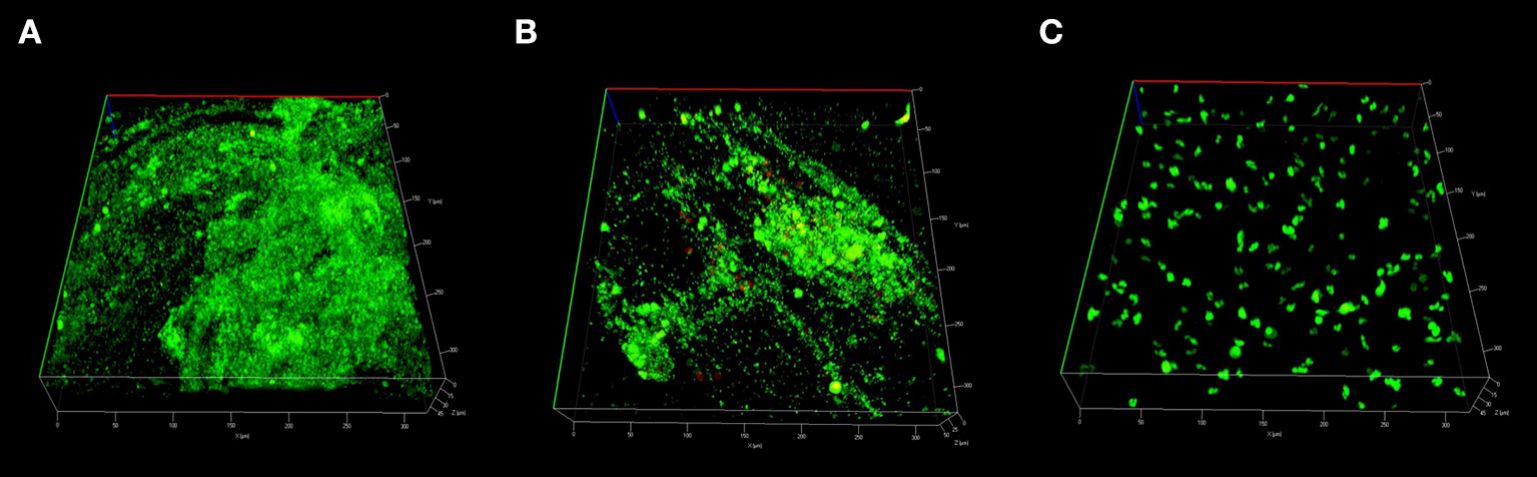
Representative CLSM images of *S. aureus* biofilms stained with LIVE/DEAD Baclight reconstructed into 3D virtual image. Small light green stains represents live bacteria, large dark green stains represents mammalian cells and large red stains represents dead mammalian cells. Sinus mucosa treated with **(A)** Twice daily saline flush (NT) showing dense population of live bacterial biofilms; **(B)** CG gel with twice-daily saline flush showing moderate population of live bacterial biofilms; **(C)** CG-DG gel with twice-daily saline flush showing no bacterial biofilms. CLSM, Confocal laser scanning microscopy; *S. aureus, Staphylococcus aureus*; NT, No treatment; CG, Chitogel; CG-DG, Chitogel- Deferiprone-Gallium Protoporphyrin.

COMSTAT2 assessment showed a significant reduction of biofilm biomass in CG-DG treated sheep compared to NT controls (*p* = 0.03, One-way ANOVA, Kruskal-Wallis test), but not between NT and CG treated sheep. Compared to no-treatment controls, CG-DG gel and CG reduced *S. aureus* biofilms by 82 and 20% respectively (Figure 4). Representative CLSM images showing LIVE/DEAD BacLight staining of *S. aureus* biofilms seen in Figure 5.

#### Histopathology analysis of sinus mucosa harvested from sheep in efficacy arm

**Figure 6:**
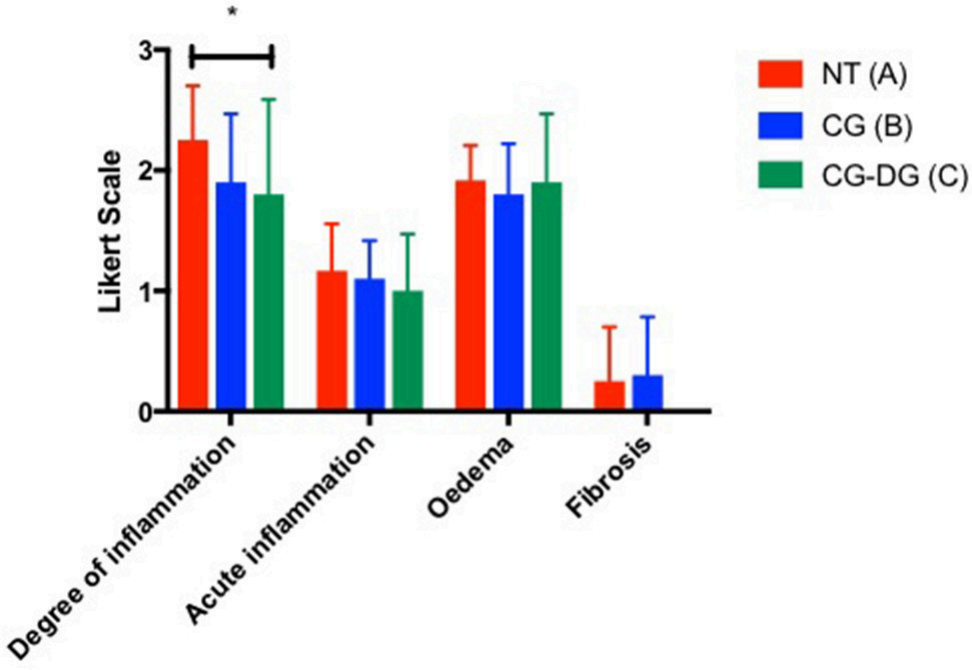
Bar graph showing degree of inflammation, acute inflammation, oedema and fibrosis grading between (A) Twice daily saline flush (NT), (B) CG gel with twice-daily saline flush, and (C) CG-DG gel with twice-daily saline flush. Significant reduction is seen in degree of inflammation of sinus mucosa between CG-DG and NT. *P < 0.05, 2-way analysis of variance (ANOVA) with Dunnett's multiple comparison test. NT, No treatment; CG, Chitogel; CG-DG, Chitogel- Deferiprone-Gallium Protoporphyrin.

There was a significant reduction in the degree of inflammation of sheep sinus mucosa between CG-DG treated group and no treatment (*p* = 0.0476, CI 95% 0.004116 to 0.8959). No significant differences were observed in degree of inflammation between CG only group and no treatment controls. Looking at acute inflammation, oedema, fibrosis and cilia, there were no significant differences in sheep sinus mucosa across all groups (Figure 6).

## Discussion

In this study we were able to show that CG-DG is safe and effective in killing *S. aureus* biofilms *in vivo* using a sheep sinusitis model described previously. The anti-inflammatory effects seen in the sinus mucosa of CG-DG group might be due to the effective eradication of biofilms.

The FDA approved oral dose of Deferiprone that is safe to use in humans is up to 75–99 mg/kg/day. Spino et al reported that following an oral dose of 1,500 mg Deferiprone (20 mg/kg) the mean maximum serum deferiprone concentration (Cmax) of non-iron-loaded healthy subjects was 20 μg/mL (Spino et al., 2015). Following one topical CG-DG application the highest plasma Deferiprone concentration measured in this study was 0.18 μg/ml, which is 110 times less than one oral dose of Deferiprone. In addition, GaPP was not detected in the plasma of any of the sheep treated with CG-DG gel. In an *in vivo* model we have to also account for some accidental oral ingestion of the sinus flushes which may reflect that the true plasma level of deferiprone might be even lower in human application as patients are instructed to apply sinus rinses head down and allow the rinses to wash out. Therefore, negligible Deferiprone plasma concentrations and the absence of GaPP in plasma, together with no observed adverse effects (e.g., no sinus mucosa damage, no ciliary denudation) indicate safety of CG-DG gel *in vivo*.

Iron is an essential element for bacterial growth, survival and replication. Deferiprone is an iron-chelator, capable of chelating free iron at the ratio 3:1 and approved by the Food and Drug Administration (FDA) for the treatment of Thalassemia Major (Olivieri et al., 1998). Deferiprone has slight anti-microbial properties by capturing iron from the environment around bacteria, causing a depletion of iron as a nutrient source (de Léséleuc et al., 2012). Deferiprone also has been shown to accelerate wound healing with enhanced skin closure after topical application *in vivo* (Mohammadpour et al., 2013). Gallium Protoporphyrin IX belongs to the family of non-iron metalloporphyrins and has antibacterial properties. The compound shows structural similarity to haem, therefore, it can mimic haem as a preferred iron source of bacteria (Stojiljkovic et al., 1999). Once inside the bacterial cell however, non-iron metalloporphyrins such as Gallium Protoporphyrin IX preserve their structure and show antibacterial effects by interfering with essential cellular pathways in the cytoplasm and in the plasma membrane causing bacterial cell death (Reniere et al., 2007). Combining Deferiprone and Gallium Protoporphyrin IX has potent synergistic antimicrobial properties against a range of bacteria including Multi Drug Resistant bacteria and Methicillin Resistant *S. aureus* (MRSA) (Richter et al., 2016). The Deferiprone and Gallium Protoporphyrin IX combination is thought to exert its anti-biofilm effects by interfering with the iron metabolism of *S. aureus* which is involved in membrane bound respiration, bacterial growth, protects against reactive oxygen species, and increases bacterial virulence factors (Braun, 2001; Weinberg, 2009).

In this study, CG gel showed the capacity to act as a drug carrier, facilitating the topical delivery of DG to biofilms in the sinonasal cavities. To exert the full anti-biofilm potential of DG it is imperative that Deferiprone is first applied followed by Gallium Protoporphyrin IX (Richter et al., 2016). Richter et al. described the quick release of hydrophilic Deferiprone from the CG gel within the first 48–72 h followed by a sustained release of hydrophobic Gallium Protoporphyrin IX reaching 20–25% over 20 days (Richter et al., 2017b) which reinforces the anti-biofilm effects of DG.

In the last decade, Chitogel has been largely used in ENT surgery to improve patient outcomes post endoscopic sinus surgery (Athanasiadis et al., 2008; Valentine et al., 2010; Ngoc Ha et al., 2013; Chung et al., 2016) due to its effective hemostatic (Klokkevold et al., 1991, 1992; Rao and Sharma, 1997; Chou et al., 2003; Pusateri et al., 2003; Valentine et al., 2009, 2010, 2011; Chung et al., 2016), wound healing (Biagini et al., 1991; Stone et al., 2000; Azad et al., 2004), anti-adhesion (Kennedy et al., 1996; Costain et al., 1997; Vlahos et al., 2001; Diamond et al., 2003; Zhou et al., 2004, 2010; Athanasiadis et al., 2008; Medina et al., 2012; Medina and Das, 2013; Cabral et al., 2015) and antimicrobial (Rhoades and Roller, 2000; No et al., 2002; Paramasivan et al., 2014a) properties and was recently FDA approved for use after sinus surgery. CG gel comprises succinyl-chitosan which is a chitosan polymer produced by the hydrolysis of chitin, found in the exoskeletons of crustaceans. Incorporating DG into CG gel strengthens the gel's anti-biofilm effects which might help improve the outcome of recalcitrant and post endoscopic sinus surgery patients.

CG-DG has been shown to have significant anti-biofilm activity not only against *S. aureus* but also MRSA, *S. epidermidis* and *P. aeruginosa* biofilms (Richter et al., 2017b). The anti-biofilm activity of DG against multiple pathogens has the added potential of treating polymicrobial infections. This broad activity makes topical CG-DG a valuable treatment alternative that can be applied within the same outpatient setting while waiting for sensitivity result to become available.

In February 2017, the World Health Organization (WHO) released a global priority list of pathogens to guide research and development of new antibiotics. Amongst the list, MRSA has been classified as a high priority pathogen and *P. aeruginosa* as critical. This also suggests that as a novel antimicrobial agent CG-DG gel has great potential for broader applications in various clinical settings.

## Conclusions

Topically applied CG-DG gel effectively reduced *S. aureus* biofilms with no observed topical and systemic adverse effects in a sheep sinusitis model, indicating safety and efficacy of CG-DG gel *in vivo*. The use of Chitogel to enhance the delivery of Deferiprone and Gallium Protoporphyrin IX offers otolaryngologists an alternative method to treat surgically recalcitrant CRS.

Clinical trials are currently underway to investigate the safety and efficacy of CG-DG gel in patients with recalcitrant chronic rhinosinusitis and in the post-operative setting.

## Author contributions

MO: project design, data collection and analysis, manuscript preparation. KR: data analysis, manuscript preparation. AD: project design, data collection. NT, CP, CJ: data analysis. SM: product manufacture and quality control. SV, AP, P-JW: project design, manuscript preparation.

### Conflict of interest statement

P-JW and SM are part of the consortium that owns the patent for Chitogel and are shareholders in the company. P-JW and SV hold a patent on the treatment combination of Deferiprone and Gallium-Protoporphyrin. The other authors declare that the research was conducted in the absence of any commercial or financial relationships that could be construed as a potential conflict of interest.

## Abbreviations

CG: Chitogel
CG-DG: Chitogel- Deferiprone-Gallium Protoporphyrin
GaPP: Gallium Protoporphyrin
SEM: scanning electron microscopy
CRS: chronic rhinosinusitis.

## References

[B1] Athanasiadis T. Beule A. G. Robinson B. H. Robinson S. R. Shi Z. Wormald P. J. (2008). Effects of a novel chitosan gel on mucosal wound healing following endoscopic sinus surgery in a sheep model of chronic rhinosinusitis. Laryngoscope 118, 1088–1094. 10.1097/MLG.0b013e31816ba576 18401274

[B2] Azad A. K. Sermsintham N. Chandrkrachang S. Stevens W. F. (2004). Chitosan membrane as a wound-healing dressing: characterization and clinical application. J. Biomed. Mater. Res. B Appl. Biomater. 69, 216–222. 10.1002/jbm.b.30000 15116411

[B3] Bendouah Z. Barbeau J. Hamad W. A. Desrosiers M. (2006). Biofilm formation by *Staphylococcus aureus* and *Pseudomonas aeruginosa* is associated with an unfavorable evolution after surgery for chronic sinusitis and nasal polyposis. Otolaryngol. Head Neck Surg. 134, 991–996. 10.1016/j.otohns.2006.03.001 16730544

[B4] Biagini G. Bertani A. Muzzarelli R. Damadei A. DiBenedetto G. Belligolli A.. (1991). Wound management with N-carboxybutyl chitosan. Biomaterials 12, 281–286. 10.1016/0142-9612(91)90035-9 1854896

[B5] Boase S. Jervis-Bardy J. Cleland E. Pant H. Tan L. Wormald P. J. (2013). Bacterial-induced epithelial damage promotes fungal biofilm formation in a sheep model of sinusitis. Int. Forum Allergy Rhinol. 3, 341–348. 10.1002/alr.21138 23307805

[B6] Braun V. (2001). Iron uptake mechanisms and their regulation in pathogenic bacteria. Int. J. Med. Microbiol. 291, 67–79. 10.1078/1438-4221-00103 11437341

[B7] Cabral J. D. McConnell M. A. Fitzpatrick C. Mros S. Williams G. Wormald P. J.. (2015). Characterization of the *in vivo* host response to a bi-labeled chitosan-dextran based hydrogel for postsurgical adhesion prevention. J. Biomed. Mater. Res. A 103, 2611–2620. 10.1002/jbm.a.35395 25545160

[B8] Chou T. C. Fu E. Wu C. J. Yeh J. H. (2003). Chitosan enhances platelet adhesion and aggregation. Biochem. Biophys. Res. Commun. 302, 480–483. 10.1016/S0006-291X(03)00173-6 12615058

[B9] Chung Y.-J. An S.-Y. Yeon J.-Y. Shim W. S. Mo J.-H. (2016). Effect of a chitosan gel on hemostasis and prevention of adhesion after endoscopic sinus surgery. Clin. Exp. Otorhinolaryngol. 9, 143–149. 10.21053/ceo.2015.00591 27090275 PMC4881319

[B10] Conly J. Johnston B. (2005). Where are all the new antibiotics? The new antibiotic paradox. Can. J. Infect. Dis. Med. Microbiol. 16, 159–160. 10.1155/2005/892058 18159536 PMC2095020

[B11] Costain D. J. Kennedy R. Ciona C. McAlister V. C. Lee T. D. (1997). Prevention of postsurgical adhesions with N,O-carboxymethyl chitosan: examination of the most efficacious preparation and the effect of N,O-carboxymethyl chitosan on postsurgical healing. Surgery 121, 314–319. 10.1016/S0039-6060(97)90360-3 9068673

[B12] Costerton J. W. (1995). Overview of microbial biofilms. J. Ind. Microbiol. 15, 137–140. 10.1007/BF01569816 8519468

[B13] de Léséleuc L. Harris G. KuoLee R. Chen W. (2012). *In vitro* and *in vivo* biological activities of iron chelators and gallium nitrate against *Acinetobacter baumannii*. Antimicrob. Agents Chemother. 56, 5397–5400. 10.1128/AAC.00778-12 22825117 PMC3457350

[B14] Diamond M. P. Luciano A. Johns D. A. Dunn R. Young P. Bieber E. (2003). Reduction of postoperative adhesions by N,O-carboxymethylchitosan: a pilot study. Fertil. Steril. 80, 631–636. 10.1016/S0015-0282(03)00759-3 12969711

[B15] Drilling A. Morales S. Boase S. Jervis-Bardy J. James C. Jardeleza C.. (2014). Safety and efficacy of topical bacteriophage and ethylened iaminetetraacetic acid treatment of *Staphylococcus aureus* infection in a sheep model of sinusitis. Int. Forum Allergy Rhinol. 4, 176–186. 10.1002/alr.21270 24449635

[B16] Ha K. R. Psaltis A. J. Tan L. Wormald P. J. (2007). A sheep model for the study of biofilms in rhinosinusitis. Am. J. Rhinol. 21, 339–345. 10.2500/ajr.2007.21.3032 17621821

[B17] Heydorn A. Nielsen A. T. Hentzer M. Sternberg C. Givskov M. Ersbøll B. K.. (2000). Quantification of biofilm structures by the novel computer program comstat. Microbiology 146, 2395–2407. 10.1099/00221287-146-10-2395 11021916

[B18] Hijazi S. Visca P. Frangipani E. (2017). Gallium-protoporphyrin IX inhibits *Pseudomonas aeruginosa* growth by targeting cytochromes. Front. Cell. Infect. Microbiol. 7:12. 10.3389/fcimb.2017.00012 28184354 PMC5266731

[B19] Illum L. Farraj N. F. Davis S. S. (1994). Chitosan as a novel nasal delivery system for peptide drugs. Pharm. Res. 11, 1186–1189. 10.1023/A:1018901302450 7971722

[B20] Kennedy R. Costain D. J. McAlister V. C. Lee T. D. (1996). Prevention of experimental postoperative peritoneal adhesions by N,O-carboxymethyl chitosan. Surgery 120, 866–870. 10.1016/S0039-6060(96)80096-1 8909523

[B21] Klinger-Strobel M. Suesse H. Fischer D. Pletz M. W. Makarewicz O. (2016). A novel computerized cell count algorithm for biofilm analysis. PLoS ONE 11:e0154937. 10.1371/journal.pone.0154937 27149069 PMC4858220

[B22] Klokkevold P. R. Lew D. S. Ellis D. G. Bertolami C. N. (1991). Effect of chitosan on lingual hemostasis in rabbits. J. Oral Maxillofac. Surg. 49, 858–863. 10.1016/0278-2391(91)90017-G 2072198

[B23] Klokkevold P. R. Subar P. Fukayama H. Bertolami C. N. (1992). Effect of chitosan on lingual hemostasis in rabbits with platelet dysfunction induced by epoprostenol. J. Oral Maxillofac. Surg. 50, 41–45. 10.1016/0278-2391(92)90194-5 1727460

[B24] Medina J. G. Das S. (2013). Sprayable chitosan/starch-based sealant reduces adhesion formation in a sheep model for chronic sinusitis. Laryngoscope 123, 42–47. 10.1002/lary.23583 23070859

[B25] Medina J. G. Steinke J. W. Das S. (2012). A chitosan-based sinus sealant for reduction of adhesion formation in rabbit and sheep models. Otolaryngol. Head Neck Surg. 147, 357–363. 10.1177/0194599812443647 22492298 PMC3685821

[B26] Mohammadpour M. Behjati M. Sadeghi A. Fassihi A. (2013). Wound healing by topical application of antioxidant iron chelators: kojic acid and deferiprone. Int. Wound J. 10, 260–264. 10.1111/j.1742-481X.2012.00971.x 22621771 PMC7950824

[B27] Nakamura K. Maitani Y. Lowman A. M. Takayama K. Peppas N. A. Nagai T. (1999). Uptake and release of budesonide from mucoadhesive, pH-sensitive copolymers and their application to nasal delivery. J. Control. Release 61, 329–335. 10.1016/S0168-3659(99)00150-9 10477805

[B28] Ngoc Ha T. Valentine R. Moratti S. Robinson S. Hanton L. Wormald P. J. (2013). A blinded randomized controlled trial evaluating the efficacy of chitosan gel on ostial stenosis following endoscopic sinus surgery. Int. Forum Allergy Rhinol. 3, 573–580. 10.1002/alr.21136 23322408

[B29] No H. K. Park N. Y. Lee S. H. Meyers S. P. (2002). Antibacterial activity of chitosans and chitosan oligomers with different molecular weights. Int. J. Food Microbiol. 74, 65–72. 10.1016/S0168-1605(01)00717-6 11929171

[B30] Olivieri N. F. Brittenham G. M. McLaren C. E. Templeton D. M. Cameron R. G. McClelland R. A.. (1998). Long-term safety and effectiveness of iron-chelation therapy with deferiprone for thalassemia major. N. Engl. J. Med. 339, 417–423. 10.1056/NEJM199808133390701 9700174

[B31] Paramasivan S. Drilling A. J. Jardeleza C. Jervis-Bardy J. Vreugde S. Wormald P. J. (2014b). Methylglyoxal-augmented manuka honey as a topical anti-*Staphylococcus aureus* biofilm agent: safety and efficacy in an *in vivo* model. Int. Forum Allergy Rhinol. 4, 187–195. 10.1002/alr.21264 24415444

[B32] Paramasivan S. Jones D. Baker L. Hanton L. Robinson S. Wormald P. J.. (2014a). The use of chitosan-dextran gel shows anti-inflammatory, antibiofilm, and antiproliferative properties in fibroblast cell culture. Am. J. Rhinol. Allergy 28, 361–365. 10.2500/ajra.2014.28.4069 25198019

[B33] Psaltis A. J. Weitzel E. K. Ha K. R. Wormald P. J. (2008). The effect of bacterial biofilms on post-sinus surgical outcomes. Am. J. Rhinol. 22, 1–6. 10.2500/ajr.2008.22.3119 18284851

[B34] Pusateri A. E. McCarthy S. J. Gregory K. W. Harris R. A. Cardenas L. McManus A. T.. (2003). Effect of a chitosan-based hemostatic dressing on blood loss and survival in a model of severe venous hemorrhage and hepatic injury in swine. J. Trauma 54, 177–182. 10.1097/00005373-200301000-00023 12544915

[B35] Rajiv S. Drilling A. Bassiouni A. James C. Vreugde S. Wormald P. J. (2015). Topical colloidal silver as an anti-biofilm agent in a *Staphylococcus aureus* chronic rhinosinusitis sheep model. Int. Forum Allergy Rhinol. 5, 283–288. 10.1002/alr.21459 25643830

[B36] Rao S. B. Sharma C. P. (1997). Use of chitosan as a biomaterial: studies on its safety and hemostatic potential. J. Biomed. Mater. Res. 34, 21–28. 8978649 10.1002/(sici)1097-4636(199701)34:1<21::aid-jbm4>3.0.co;2-p

[B37] Reniere M. L. Torres V. J. Skaar E. P. (2007). Intracellular metalloporphyrin metabolism in *Staphylococcus aureus*. Biometals 20, 333–345. 10.1007/s10534-006-9032-0 17387580

[B38] Rhoades J. Roller S. (2000). Antimicrobial actions of degraded and native chitosan against spoilage organisms in laboratory media and foods. Appl. Environ. Microbiol. 66, 80–86. 10.1128/AEM.66.1.80-86.2000 10618206 PMC91788

[B39] Richter K. Ramezanpour M. Thomas N. Prestidge C. A. Wormald P. J. Vreugde S. (2016). Mind “De GaPP”: *in vitro* efficacy of deferiprone and gallium-protoporphyrin against *Staphylococcus aureus* biofilms. Int. Forum Allergy Rhinol. 6, 737–743. 10.1002/alr.21735 26919404

[B40] Richter K. Thomas N. Claeys J. McGuane J. Prestidge C. A. Coenye T.. (2017b). A topical hydrogel with deferiprone and gallium-protoporphyrin targets bacterial iron metabolism and has antibiofilm activity. Antimicrob. Agents Chemother. 61:e00481-17. 10.1128/AAC.00481-17 28396543 PMC5444117

[B41] Richter K. Thomas N. Zhang G. Prestidge C. A. Coenye T. Wormald P.-J.. (2017c). Deferiprone and gallium-protoporphyrin have the capacity to potentiate the activity of antibiotics in *Staphylococcus aureus* small colony variants. Front. Cell. Infect. Microbiol. 7:280. 10.3389/fcimb.2017.00280 28690982 PMC5479885

[B42] Richter K. Van den Driessche F. Coenye T. (2017a). Innovative approaches to treat *Staphylococcus aureus* biofilm-related infections. Essays Biochem. 61, 61–70. 10.1042/EBC20160056 28258230

[B43] Singhal D. Foreman A. Jervis-Bardy J. Wormald P. J. (2011). *Staphylococcus aureus* biofilms: nemesis of endoscopic sinus surgery. Laryngoscope 121, 1578–1583. 10.1002/lary.21805 21647904

[B44] Singhal D. Jekle A. Debabov D. Wang L. Khosrovi B. Anderson M.. (2012). Efficacy of NVC-422 against *Staphylococcus aureus* biofilms in a sheep biofilm model of sinusitis. Int. Forum Allergy Rhinol. 2, 309–315. 10.1002/alr.21038 22434724

[B45] Singhal D. Psaltis A. J. Foreman A. Wormald P. J. (2010). The impact of biofilms on outcomes after endoscopic sinus surgery. Am. J. Rhinol. Allergy 24, 169–174. 10.2500/ajra.2010.24.3462 20537281

[B46] Spino M. Connelly J. Tsang Y.-C. Fradette C. Tricta F. (2015). Deferiprone pharmacokinetics with and without iron overload and in special patient populations. Blood 126, 3365–3365.

[B47] Stojiljkovic I. Kumar V. Srinivasan N. (1999). Non-iron metalloporphyrins: potent antibacterial compounds that exploit haem/Hb uptake systems of pathogenic bacteria. Mol. Microbiol. 31, 429–442. 10.1046/j.1365-2958.1999.01175.x 10027961

[B48] Stone C. A. Wright H. Clarke T. Powell R. Devaraj V. S. (2000). Healing at skin graft donor sites dressed with chitosan. Br. J. Plast. Surg. 53, 601–606. 10.1054/bjps.2000.3412 11000077

[B49] Valentine R. Athanasiadis T. Moratti S. Hanton L. Robinson S. Wormald P. J. (2010). The efficacy of a novel chitosan gel on hemostasis and wound healing after endoscopic sinus surgery. Am. J. Rhinol. Allergy 24, 70–75. 10.2500/ajra.2010.24.3422 20109331

[B50] Valentine R. Athanasiadis T. Moratti S. Robinson S. Wormald P. J. (2009). The efficacy of a novel chitosan gel on hemostasis after endoscopic sinus surgery in a sheep model of chronic rhinosinusitis. Am. J. Rhinol. Allergy 23, 71–75. 10.2500/ajra.2009.23.3266 19379616

[B51] Valentine R. Boase S. Jervis-Bardy J. Dones Cabral J. D. Robinson S. Wormald P. J. (2011). The efficacy of hemostatic techniques in the sheep model of carotid artery injury. Int. Forum Allergy Rhinol. 1, 118–122. 10.1002/alr.20033 22287330

[B52] Vlahos A. Yu P. Lucas C. E. Ledgerwood A. M. (2001). Effect of a composite membrane of chitosan and poloxamer gel on postoperative adhesive interactions. Am. Surg. 67, 15–21. 11206889

[B53] Weinberg E. D. (2009). Iron availability and infection. Biochim. Biophys. Acta 1790, 600–605. 10.1016/j.bbagen.2008.07.002 18675317

[B54] World Health Organization (2016). Antibiotic Resistance. Media Centre. World Health Organization(Fact Sheets).

[B55] World Health Organization (2017). Antibacterial agents in clinical development. an analysis of the antibacterial clinical development pipeline, including tuberculosis, in Medicines and Health Products Aow, Rational Use of Medicines (World Health Organization), 48.

[B56] Zhou J. Elson C. Lee T. D. (2004). Reduction in postoperative adhesion formation and re-formation after an abdominal operation with the use of N, O - carboxymethyl chitosan. Surgery 135, 307–312. 10.1016/j.surg.2003.07.005 14976481

[B57] Zhou J. Lee J. M. Jiang P. Henderson S. Lee T. D. (2010). Reduction in postsurgical adhesion formation after cardiac surgery by application of N,O-carboxymethyl chitosan. J. Thorac. Cardiovasc. Surg. 140, 801–806. 10.1016/j.jtcvs.2009.11.030 20176369

